# Supplementary material for: Internal uncertainty impacts social information use in risky choice across adolescence
Source: Commun Psychol. 2025 Sep 16;3:137. doi: 10.1038/s44271-025-00314-6 (PMC12441144; doi:10.1038/s44271-025-00314-6)

Internal uncertainty impacts social information use in risky choice across adolescence.

Supporting Information

**Figure S1** Age distribution and puberty stage, with our (assessed with the puberty development scale) of our sample. The two panels show the participants self-reported sex.

**Table S1:** Results of the regression model testing our preregistered hypotheses, using age-bins 10-12 (reference); 12-17 (“adolescents”); >18 (young adults) instead of continuous age predictors. We predicted risky decisions on each trial using predictors in the first row using a random intercept for participants. The second row denotes the odds ratio (exp of regression weight), and the second row denotes its 95% credible interval.

|  | **ChooseRisk** | |
| --- | --- | --- |
| *Predictors* | *Odds Ratios* | *CI (95%)* |
| Intercept | 1.53 | 0.97 – 2.39 |
| EV | 17.90 | 14.89 – 21.61 |
| Agegroup1 | 0.73 | 0.41 – 1.34 |
| Agegroup2 | 0.48 | 0.25 – 0.94 |
| OtherChoseRisk20 | 0.85 | 0.65 – 1.11 |
| OtherChoseRisk21 | 1.37 | 1.14 – 1.65 |
| DFE1DFD0: DFE1DFD01 | 1.08 | 0.91 – 1.28 |
| cftScore | 0.89 | 0.56 – 1.46 |
| numbersTotal | 1.27 | 0.74 – 2.20 |
| EV:Agegroup1 | 4.73 | 3.60 – 6.20 |
| EV:Agegroup2 | 10.62 | 7.66 – 14.85 |
| OtherChoseRisk20:DFE1DFD01 | 0.53 | 0.36 – 0.77 |
| OtherChoseRisk21:DFE1DFD01 | 0.96 | 0.74 – 1.25 |
| Agegroup1:OtherChoseRisk20 | 1.44 | 1.02 – 2.03 |
| Agegroup2:OtherChoseRisk20 | 1.55 | 1.06 – 2.26 |
| Agegroup1:OtherChoseRisk21 | 0.88 | 0.69 – 1.13 |
| Agegroup2:OtherChoseRisk21 | 1.06 | 0.81 – 1.40 |
| Agegroup1:DFE1DFD01 | 0.78 | 0.62 – 0.97 |
| Agegroup2:DFE1DFD01 | 0.64 | 0.49 – 0.82 |
| Agegroup1:OtherChoseRisk20:DFE1DFD01 | 1.51 | 0.93 – 2.48 |
| Agegroup2:OtherChoseRisk20:DFE1DFD01 | 1.72 | 1.01 – 2.95 |
| Agegroup1:OtherChoseRisk21:DFE1DFD01 | 1.05 | 0.74 – 1.47 |
| Agegroup2:OtherChoseRisk21:DFE1DFD01 | 1.19 | 0.80 – 1.76 |
| **Random Effects** | | |
| σ^2^ | 3.29 | |
| τ_00_ _participant_ | 1.87 | |
| ICC | 0.36 | |
| N _participant_ | 160 | |
| Observations | 23025 | |
| Marginal R^2^ / Conditional R^2^ | 0.364 / 0.452 | |


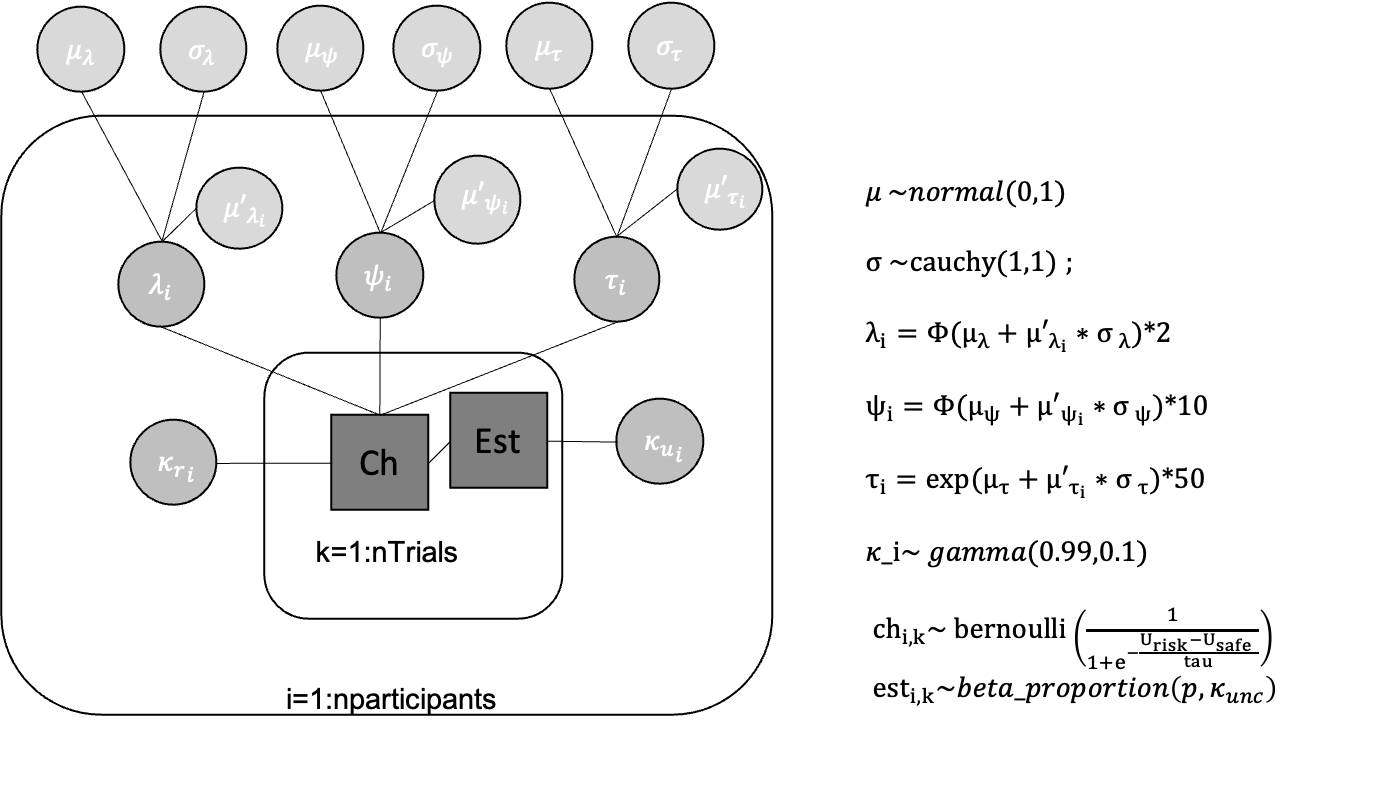


**Figure S2:** Graphical representation of the Bayesian utility model. Hierarchy of parameters depend on subjects and condition. Subject level and hyperprior means were sampled from unit normal distribution and transformed into their actual values that were fed into the modelling equation using non-centered parametrization in order to achieve efficient subject level parameter sampling. Φ denotes the unit normal cumulative distribution function that was used to keep parameters within their boundaries.


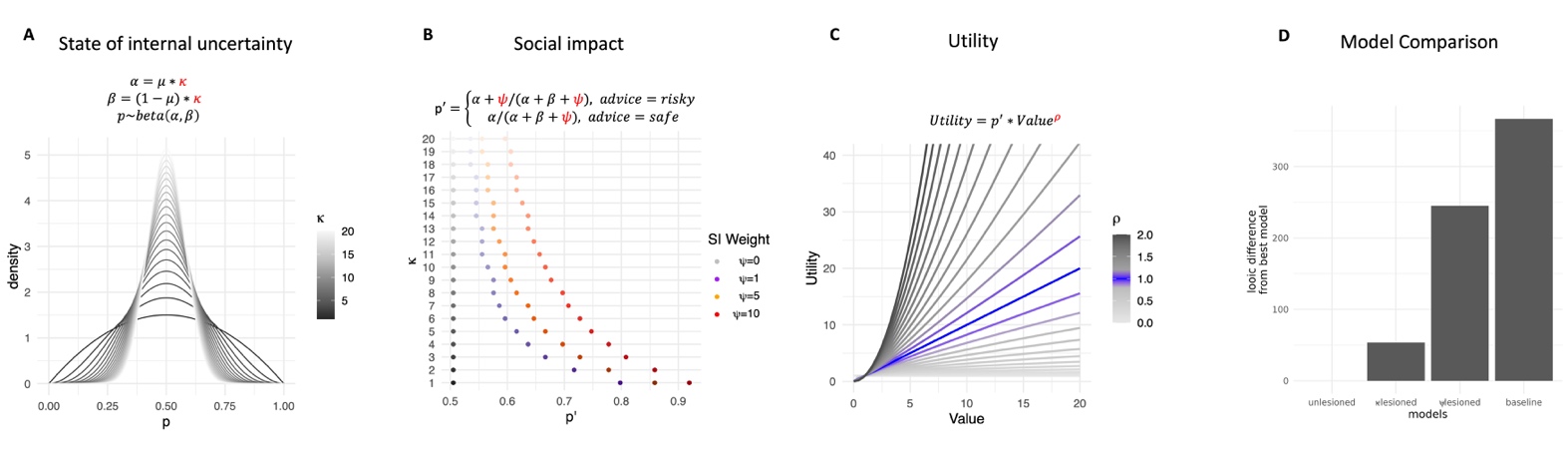


**Figure S3.** Alternative depiction of the model and its components in A, B and C and model comparison using looic in D.

# Parameter Recovery

To establish that the parameters modes of the uncertain utility model are identifiable we re-fit the uncertain utility model to its own posterior predictions by simulating responses from full combinations with 10 equally spaced parameter values with following parameter boundaries: $\lambda\epsilon\left\{ 0.1\ldots2 \right\}, \kappa_{unc/risk} \epsilon\left\{ 1\ldots20 \right\}, \psi\epsilon\left\{ 1\ldots8 \right\},$. $\tau\epsilon\left\{ 1\ldots20 \right\}$

Fitting of the parameter recovery was done using the L-BFGS algorithm for optimizing posterior modes in stan. Each value was varied whereas the others were kept constant at values $\lambda=1, \kappa_{unc/risk}=9, \psi_{risk/safe}= 1,$. $\tau=0.1$. The simulation and fitting cycle were repeated 200 times for each varied value.

**Figure S4:** Generative parameters are plotted on the x-axes and the parameter estimates obtained from fitting the model to the synthetic data are plotted on the y-axes. Red: mean recovered parameter values with bootstrapped 95% confidence intervals of the mean as error bars (covered by the dots). The dark blue line shows linear fit. Results from individual recovery runs are shown as half-transparent black dots. The black line is a diagonal on which one would expect to find parameters that recover perfectly.

**Figure S5:** Parameter correlations. The values in the cells and colour show the Pearson correlation between parameters in rows and columns. Rows denote parameters obtained from fitting the model to choices simulated from the model under varying the generative parameters in the columns. Diagonal elements illustrate the high correlation between simulated and recovered parameters. Spurious correlations are generally lower (off diagonal). A notable exception is the rate parameter for decisions under risk, that is trading off with other parameters to some extent.

# Alternative Models

It is possible that a simpler version of the model full model presented in Eq 5-7 of the manuscript is sufficient to explain the choices of our participants. Using model comparison, we therefore test less-complex hypotheses about the nature of social influence by lesioning away parameters of the full model. For instance, substituting Eq 3 for Eq 5, results in a baseline model in which social information has no effect. Using Eq 4 instead of Eq 5 to compute $p^{'}$ ignores $\psi$ and results in a model insensitive to variations in social information use, but in which social impact still depends on how unsure participants are about probabilities.

Additionally to the lesioned version of the model explained in the main text we added three other models in our model comparison, akin to those described in Ciranka and van den Bos (2019). As an alternative to Bayesian updating, lesioning the effect of $\kappa$*,* advice could impact utilities directly, without being sensitive to how unsure they are. This can be achieved by an “other conferred utility” (OCU) (Chung et al., 2015; Ciranka & van den Bos, 2019) model of social influence in risky choice, where advice directly shifts the participants' representation of the utility of the option that was advised, by adding SI to Eq 6 and using Eq 3 to compute outcome probabilities.

|  | $\mathrm{EU}_{risk}=p*U_{risk}+SI_{risk}*\Psi_{risk},$  $\mathrm{EU}_{safe}=U_{safe}+SI_{safe}*\Psi_{safe}.$ | (S1) |
| --- | --- | --- |

Another possibility is that a social context makes adolescents more sensitive towards rewards. We formalize this view by invoking a strictly positive, free parameter on the “reward sensitivity” part of each participants’ utility function, that occurs when social information is present:

|  | $U_{\mathrm{Social}}=V_{\mathrm{social}}^{\lambda}+\psi$ | (S2) |  |
| --- | --- | --- | --- |

Increased distraction has also been discussed as a mechanism determining adolescent decision making under uncertainty. Therefore, we introduce another model which augments the probability to choose risk with a “trembling hand” error term modelling an additional probability of guessing in a social context

|  | $p_{choose\_risk\_social}\sim Bernoulli\left( (1-\zeta)*\pi+\frac{\zeta}{2} \right)$. | (S3) |  |
| --- | --- | --- | --- |

The priors on $\psi$ in the reward sensitivity model were:

$$\mu_{\psi}\sim normal(0,1)$$

$$\mu_{i_{\psi}}^{'}\sim normal(0,1)$$

$$\sigma_{\psi}\sim gamma(1,0.1)$$

The prior for $\zeta$ in the distraction (“trembling hand”) model was:

$$\zeta_{i}\sim beta(1, 1)$$

To establish whether the models can be distinguished using model comparison, we simulated, for each participant and model, 100 experiment runs using the individuals’ empirical parameter estimates under the respective model. We then fitted the generated datasets with each model and evaluated, using approximate leave one out cross validation (Vehtari, Gelman, and Gabry 2015) how often the data generating model provided the best out of sample prediction of the each participant after fitting it to the synthetic data. If models fitted equally well, we selected a winner among them at random. We then estimated the conditional probability that a model fits best given the true generative model (p(fit|gen), figure S5a). Another metric more critical for evaluating our empirical results is p(gen|fit) (figure S5b), which is the probability that the data was generated by a specific model, given that the model was observed as providing the best fit to the generated data. We compute this probability using Bayes’s theorem, with a uniform prior over models (p(gen)). We note that a parsimonious model of Bayesian learning without additional social parameters (unc only) trades off substantially with all other models.

**Figure S6** Model recovery. The models were generally distinguished with above chance probability and are more likely to be identified as the data generating model than their alternatives as indicated bigh higher diagonal than off-diagnoal values. Columns and rows show models that we fitted to the data and those that generated the data the models were fitted to. Colours denote the confusion (a, rows add to 1) and inversion (b, columns add to 1) probability and diverge from yellow to blue or green at 1/6, which is the probability of a model picked at random providing the best fit.

## Age trends in model parameters

**Figure S7**. Regression weights (x-axes) resulting from predicting the model parameters (panels) with linear and quadratic age (y axis). The vertical line denotes a null effect. The boxplots show the full posterior regression weights and values the posterior mean. Blue colours are effects that are on average positive, red negative.

## EV interaction regression


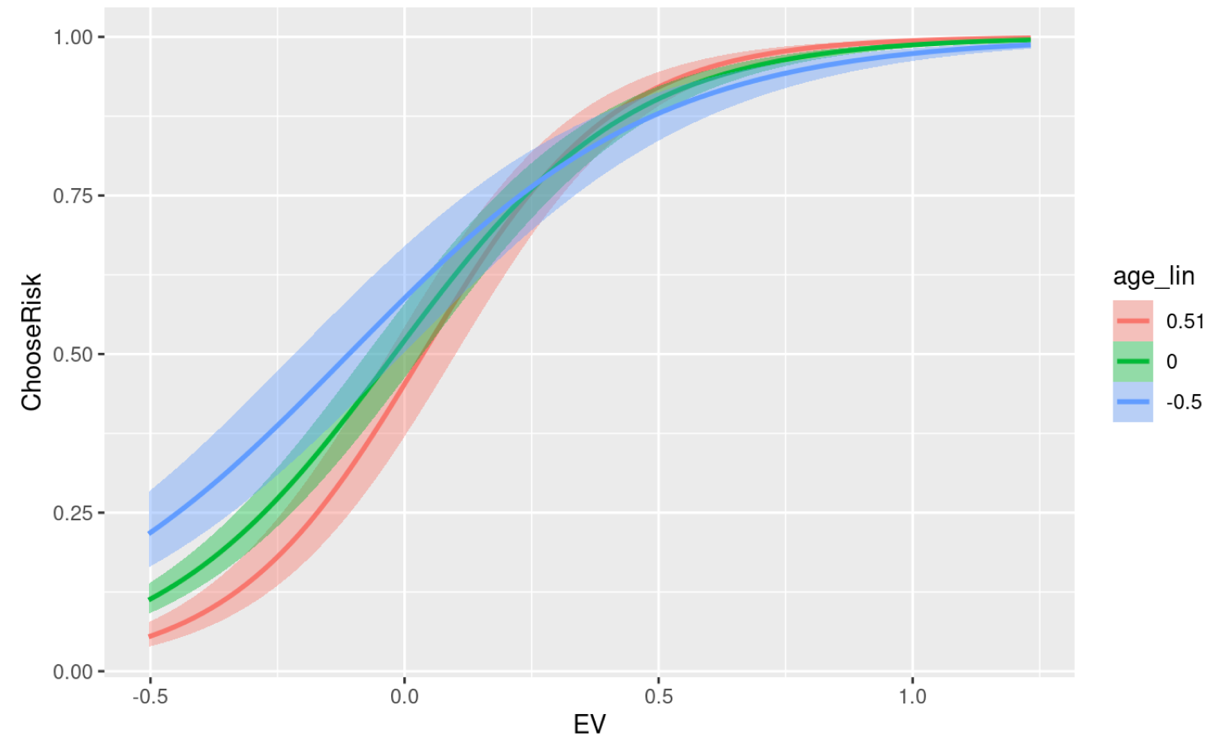


**Figure S8.** Marginal effects of the interaction between expected value (x-axis) and age (colors) on risky choice (y-axis). Younger people (blue) choose more risky than older people when expected values are low, and less risky when they are high.

**References**

Ciranka, Simon, and Wouter van den Bos. 2019. “Social Influence in Adolescent Decision-Making: A Formal Framework.” *Frontiers in Psychology* 10 (August): 1915.

Vehtari, Aki, Andrew Gelman, and Jonah Gabry. 2015. “Practical Bayesian Model Evaluation Using Leave-One-out Cross-Validation and WAIC.” *ArXiv [Stat.CO]*, July. https://arxiv.org/abs/1507.04544.

Screenshots from the Instructions

1.


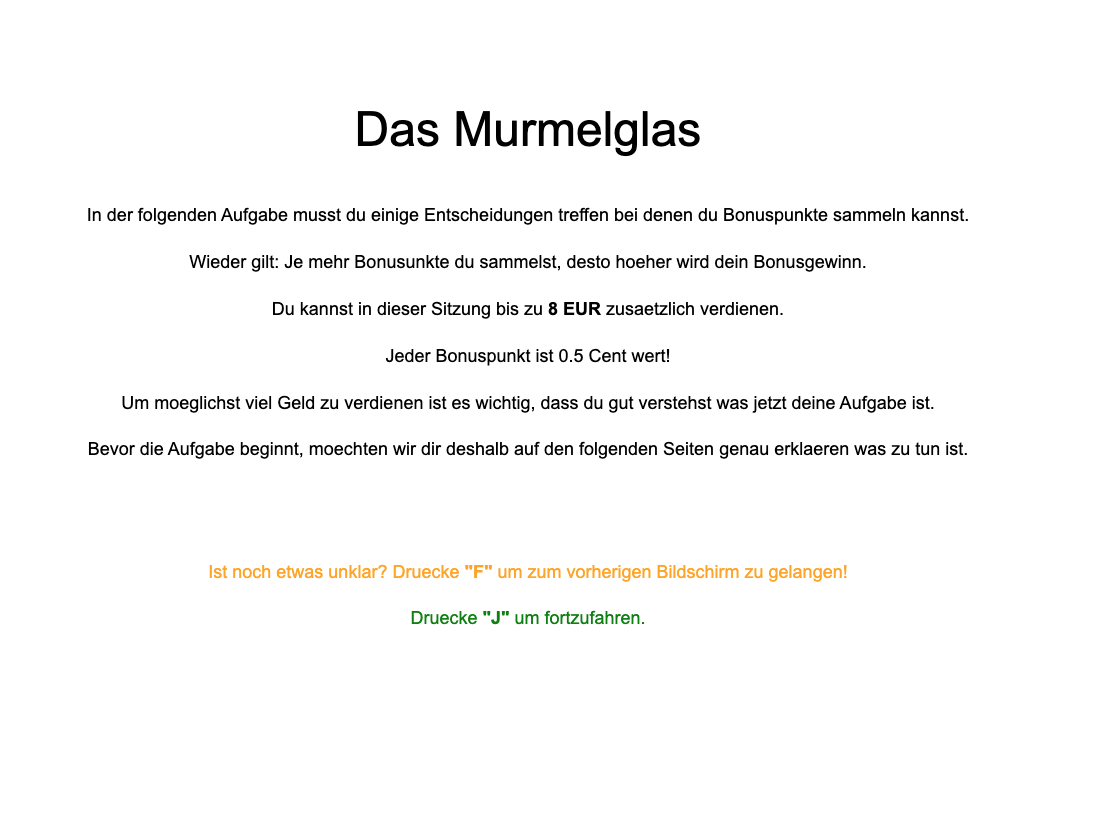


2.


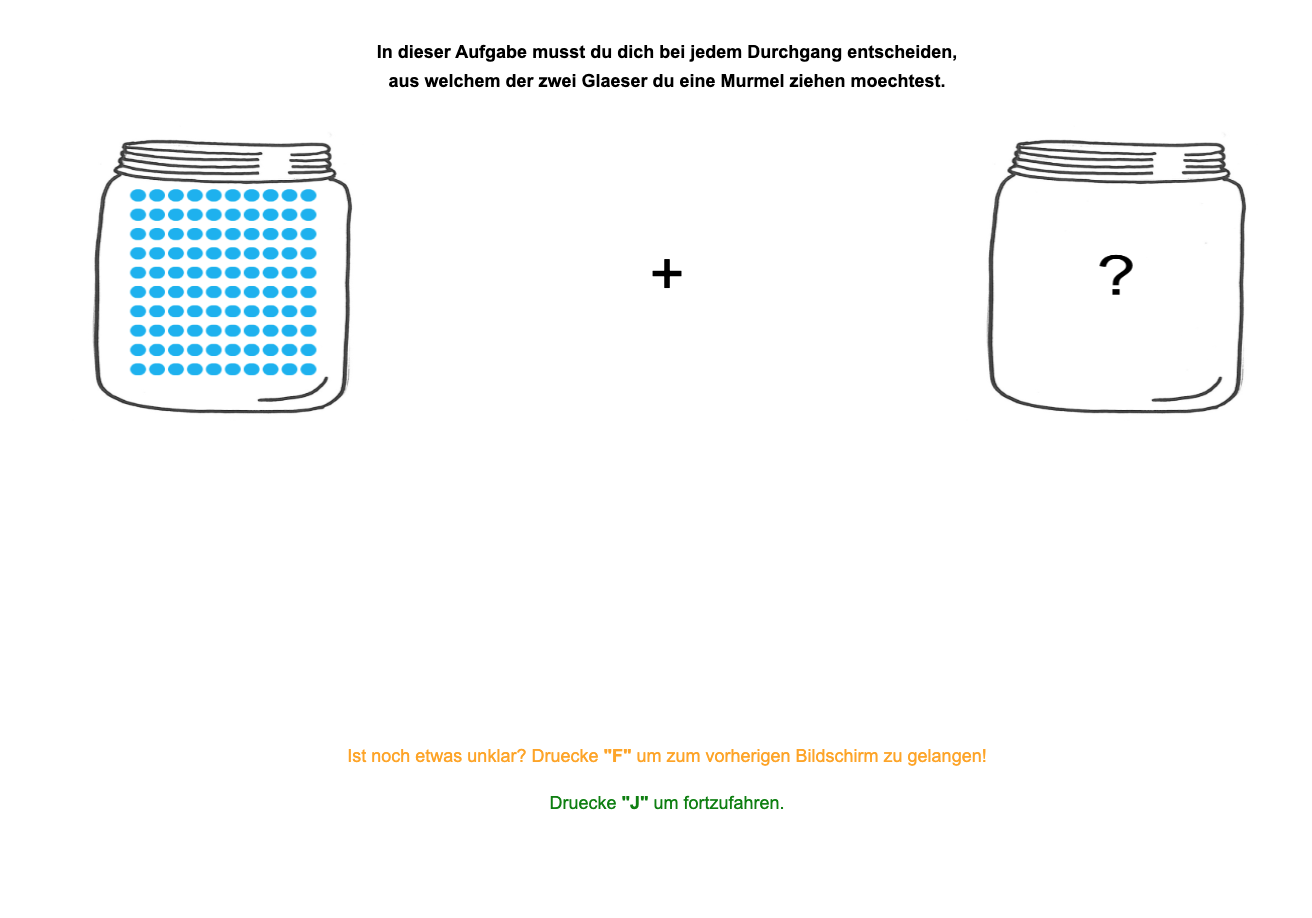


3.


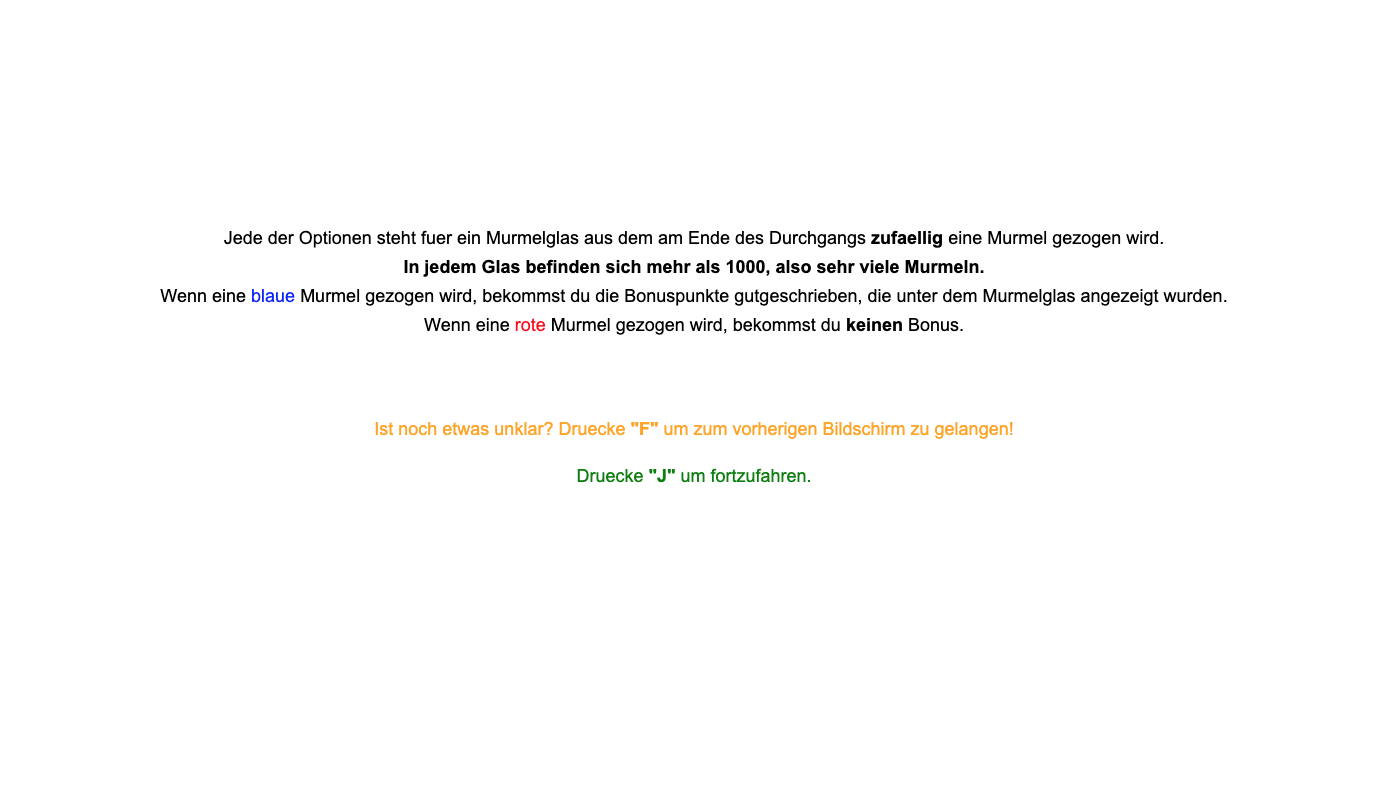


4.


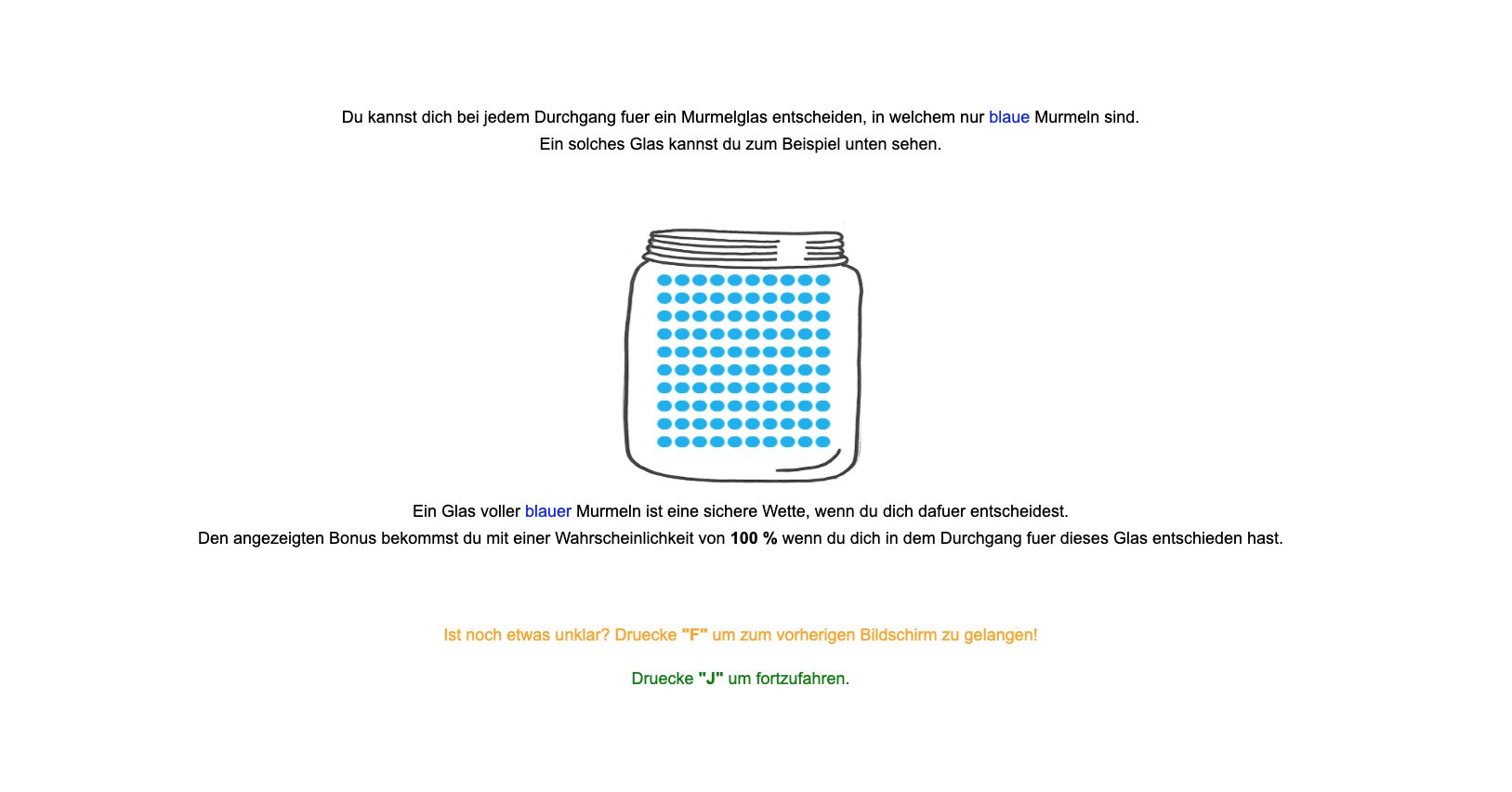


5.


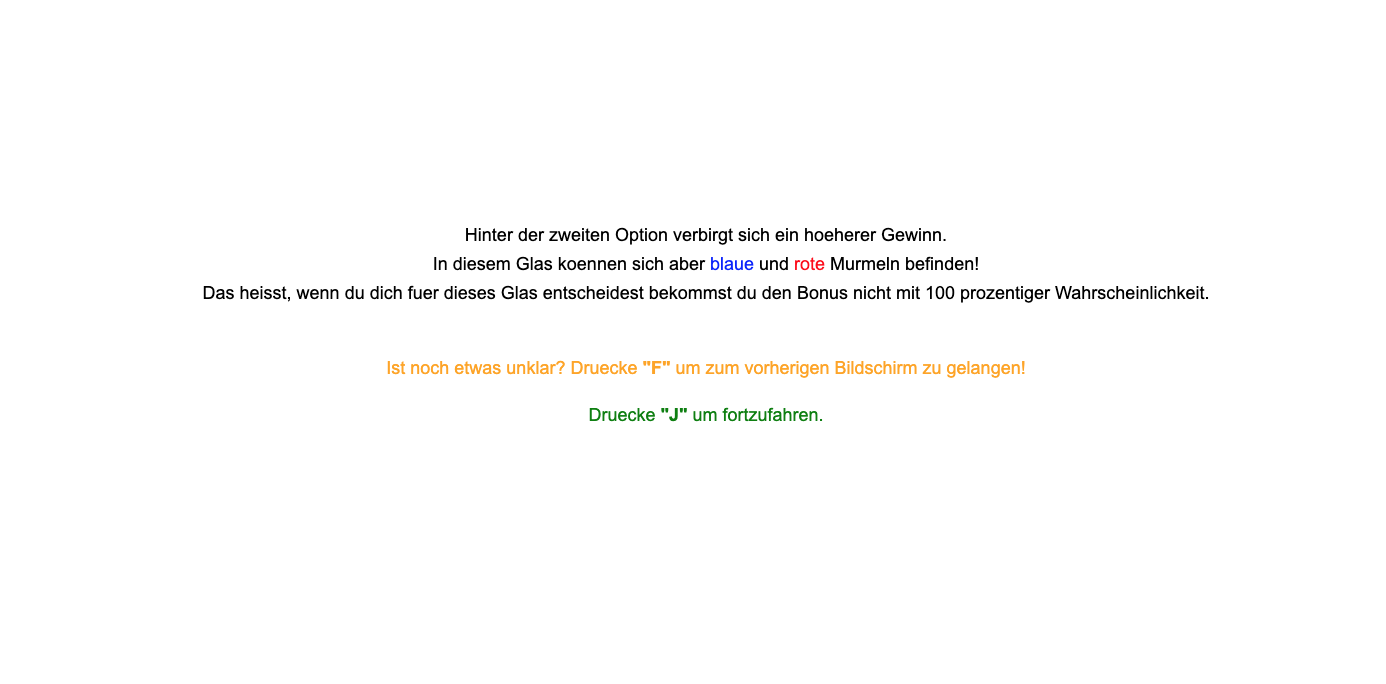


6.


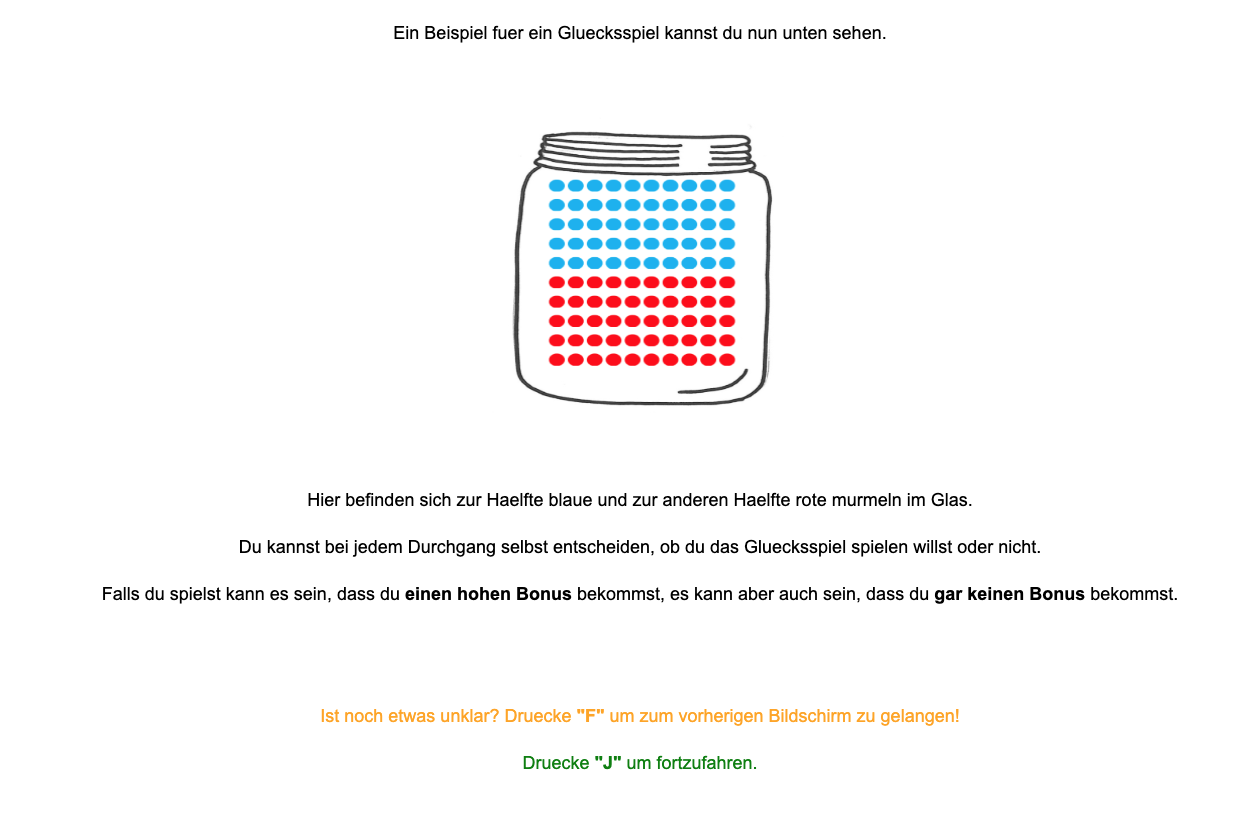


7.


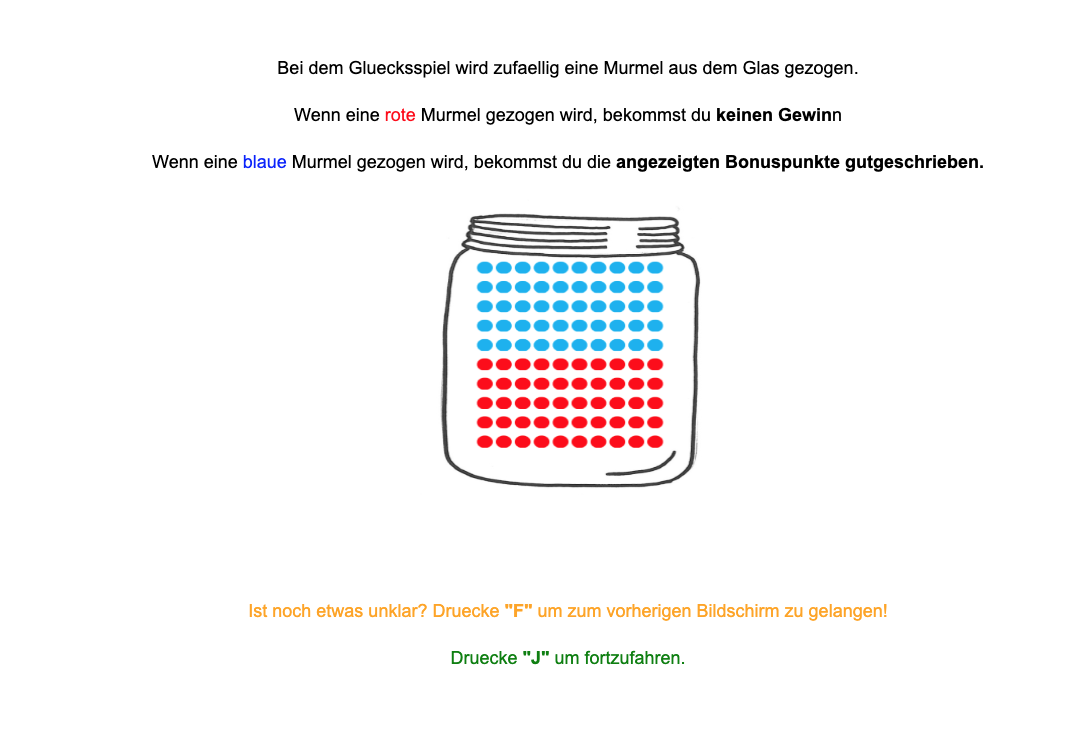


8.


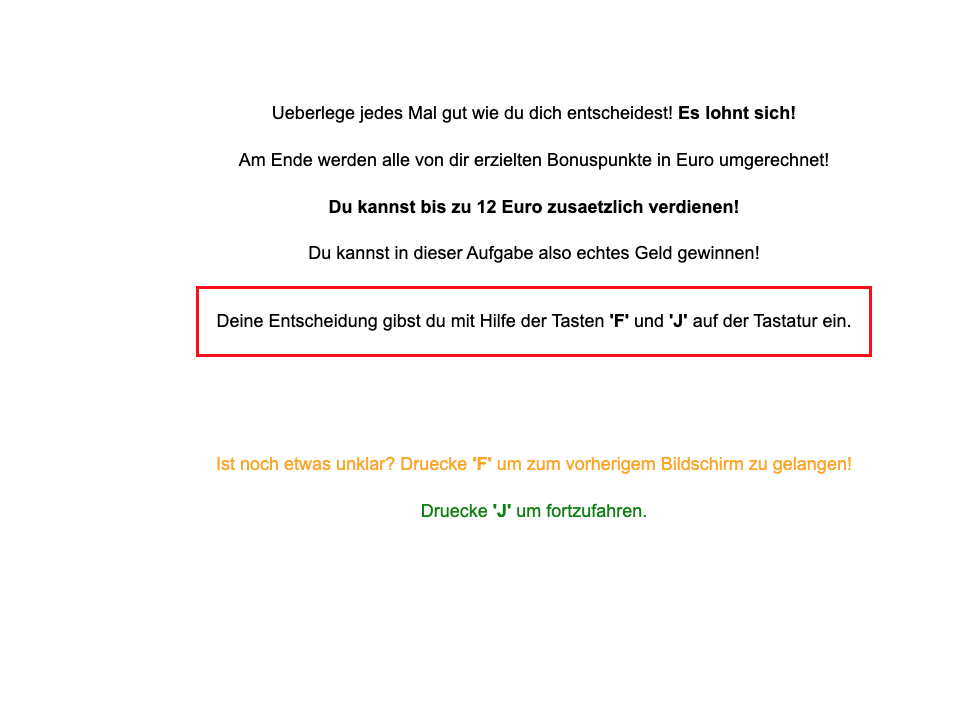


Comprehension Check 1


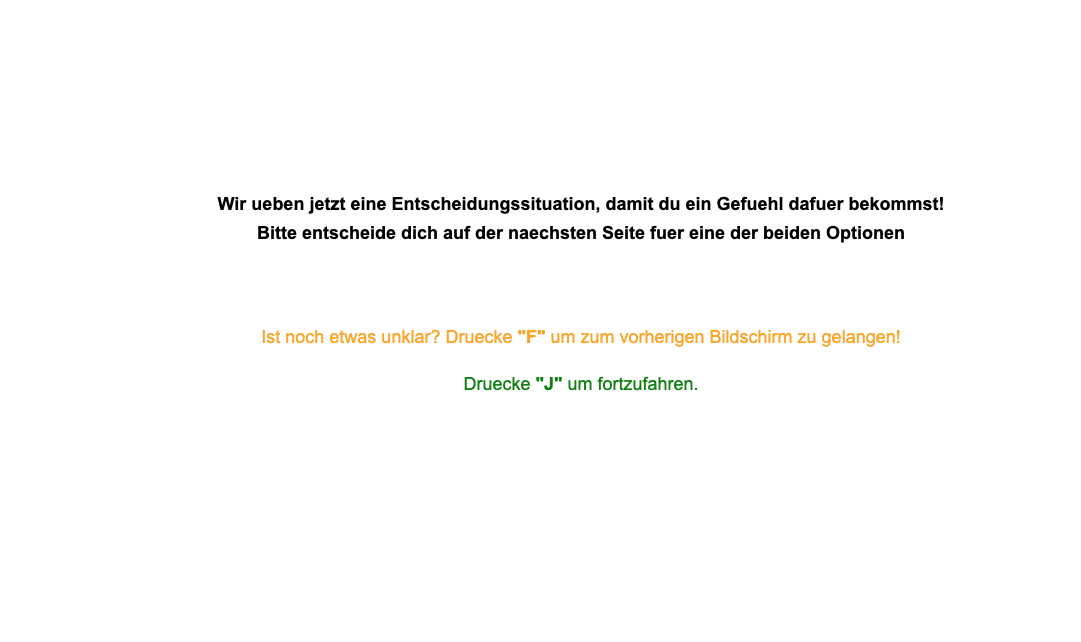


9.


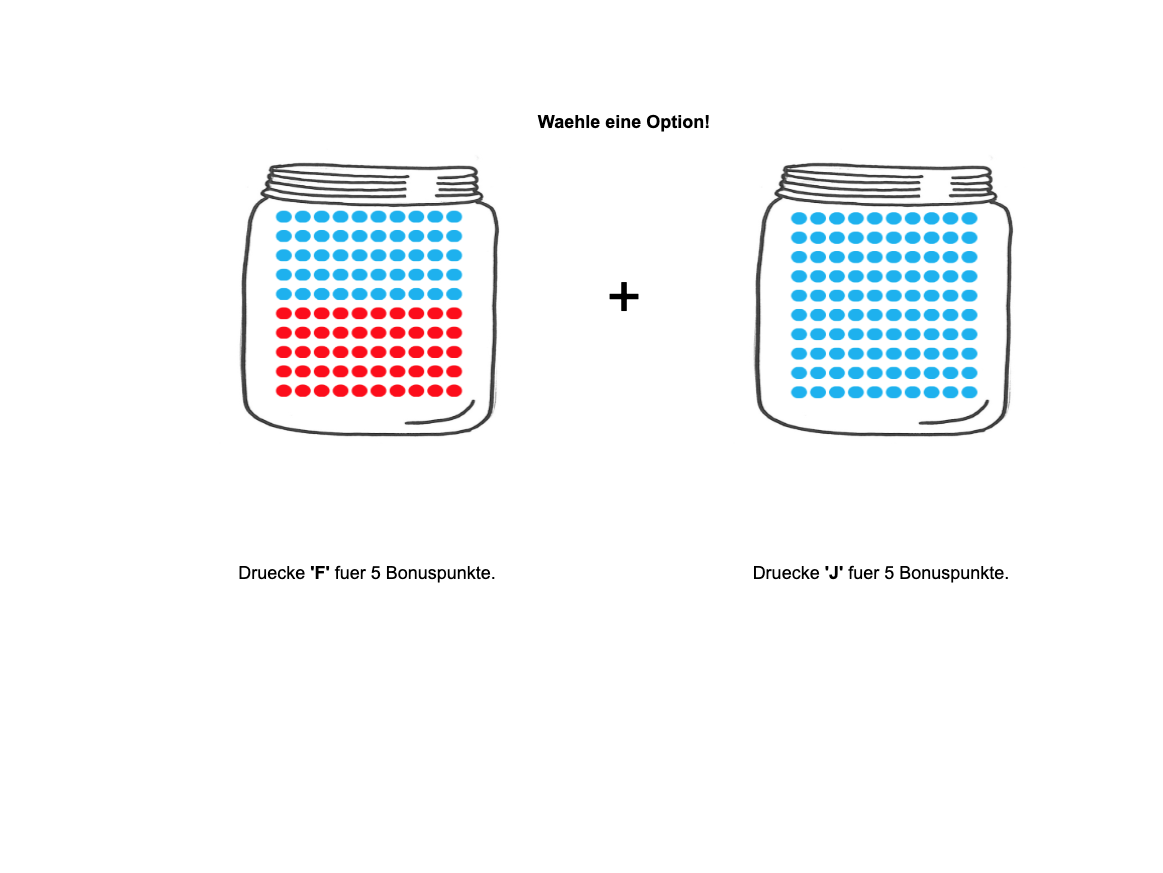


If wrong (10a and then back to 9):


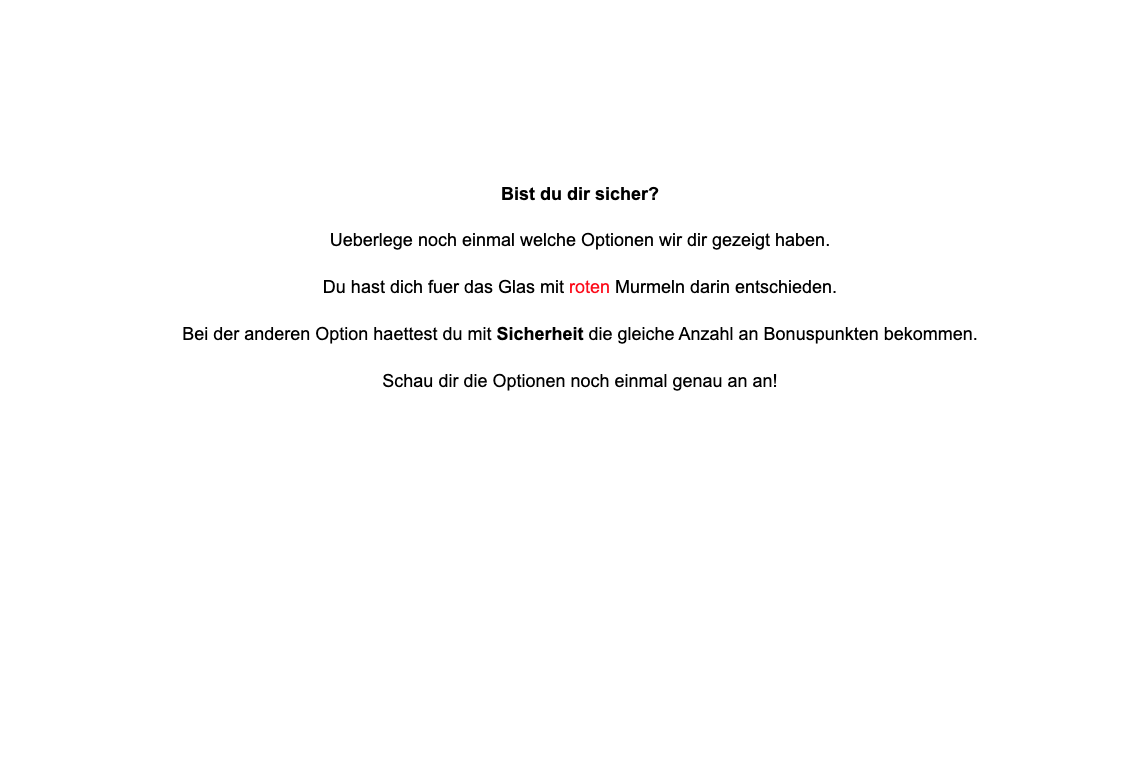


If correct (10b):


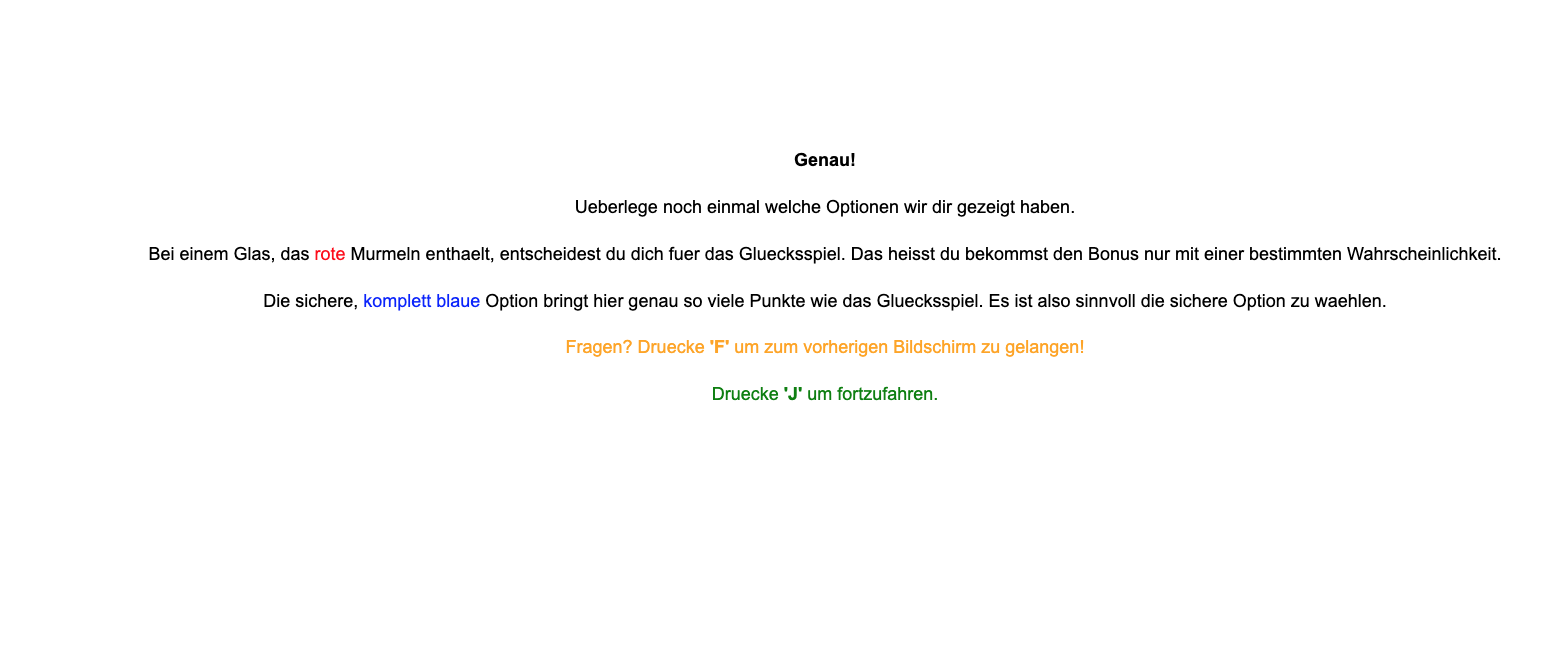


11.


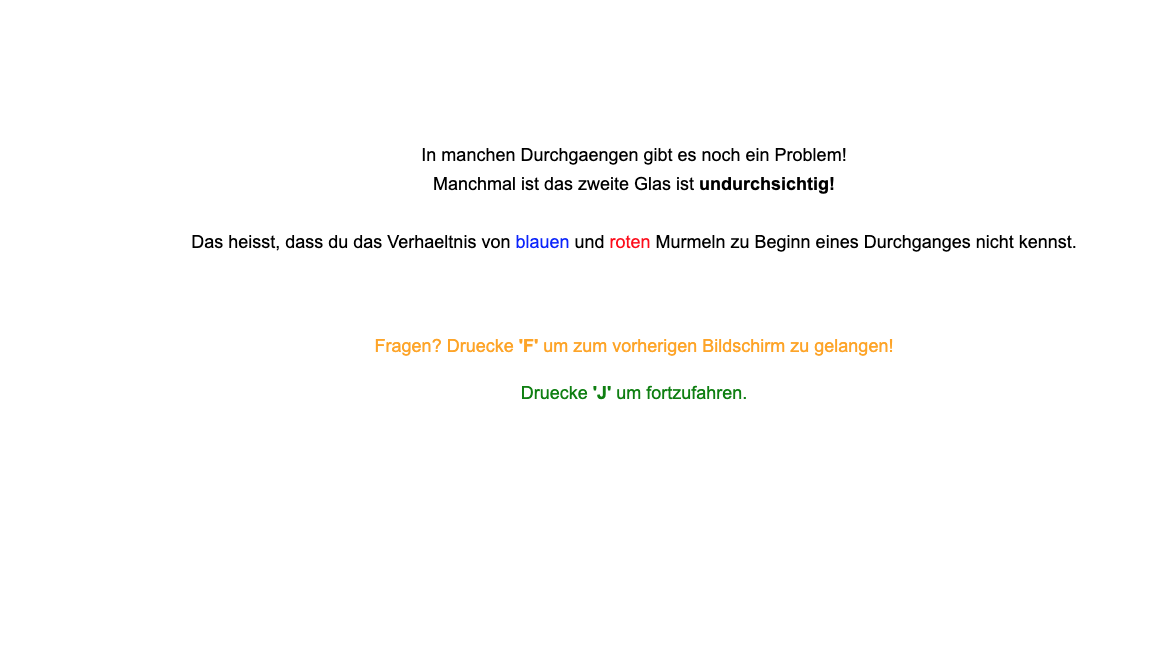


12.


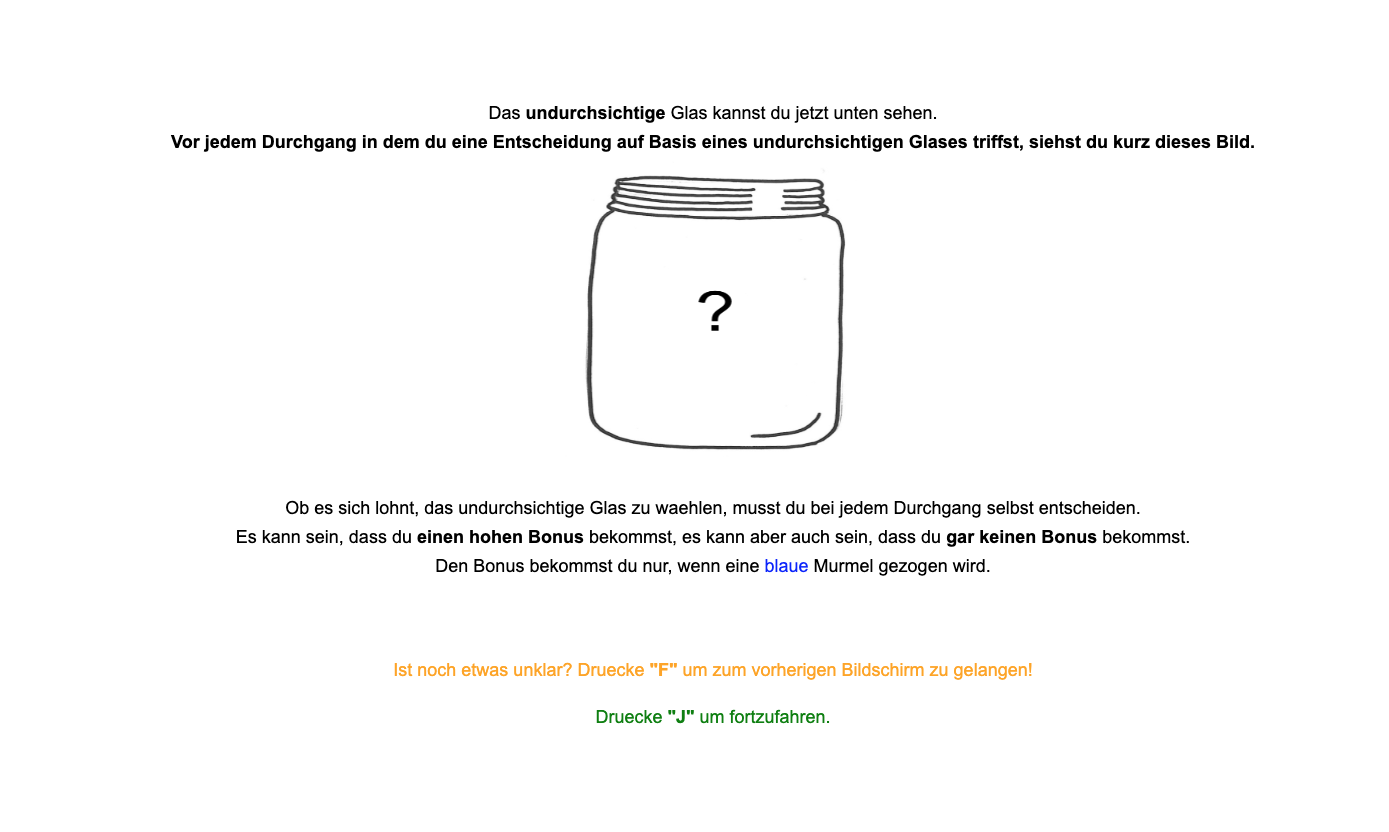


13.


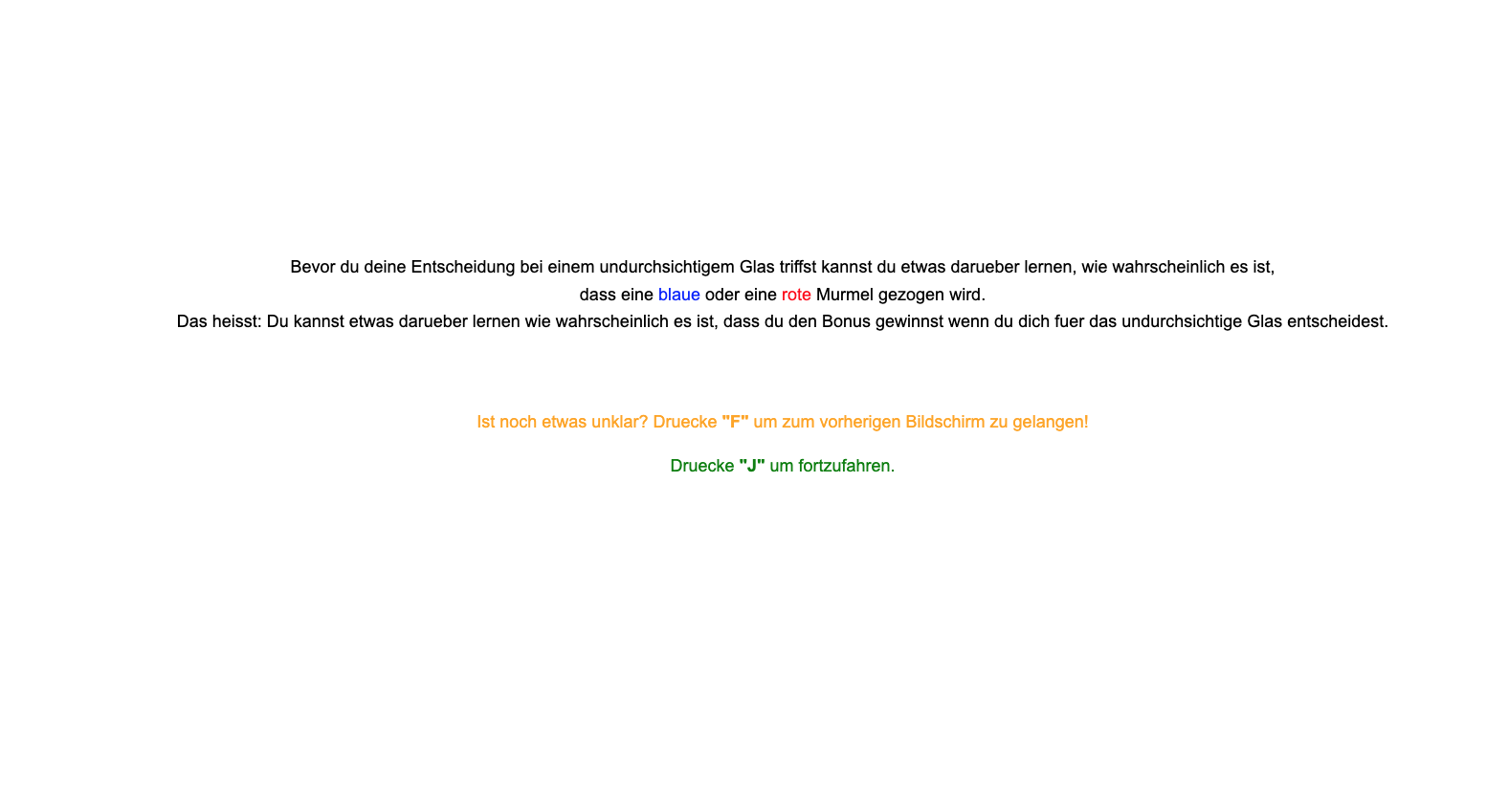


14.


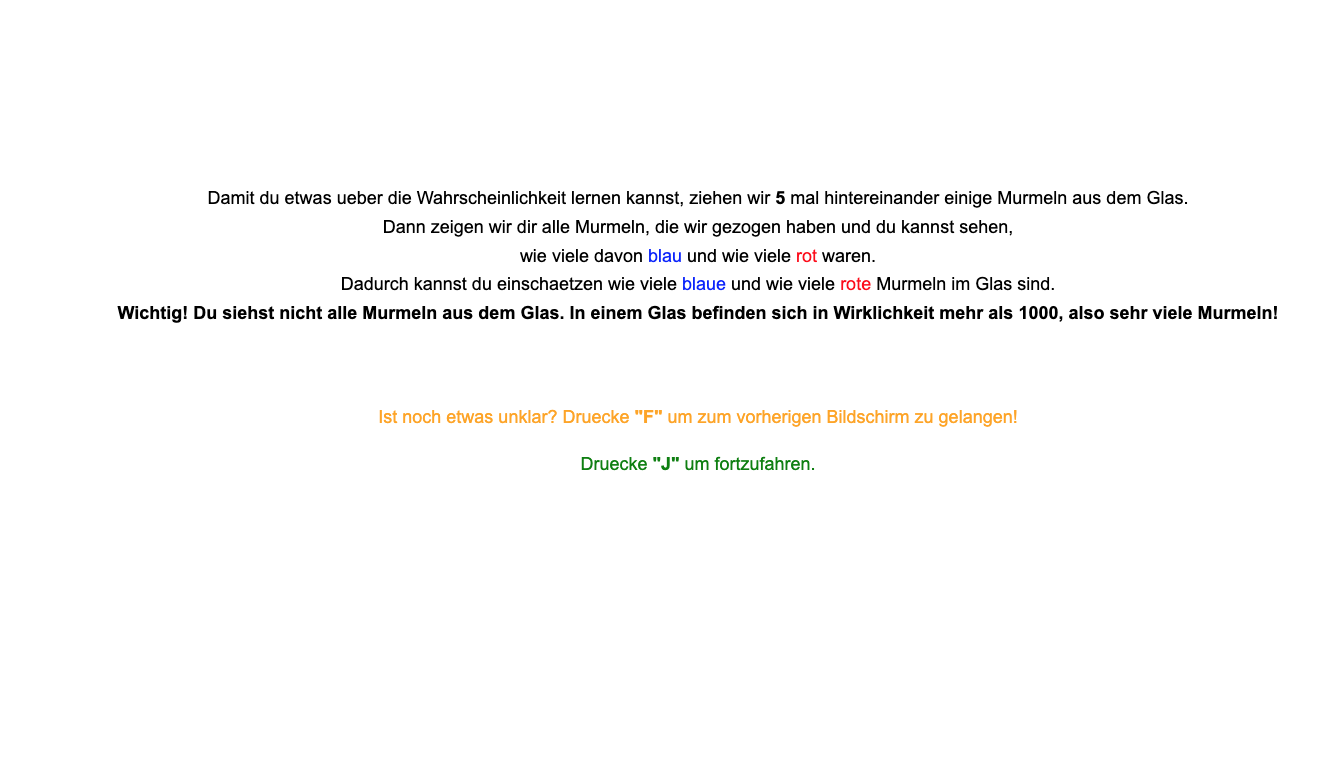


15.


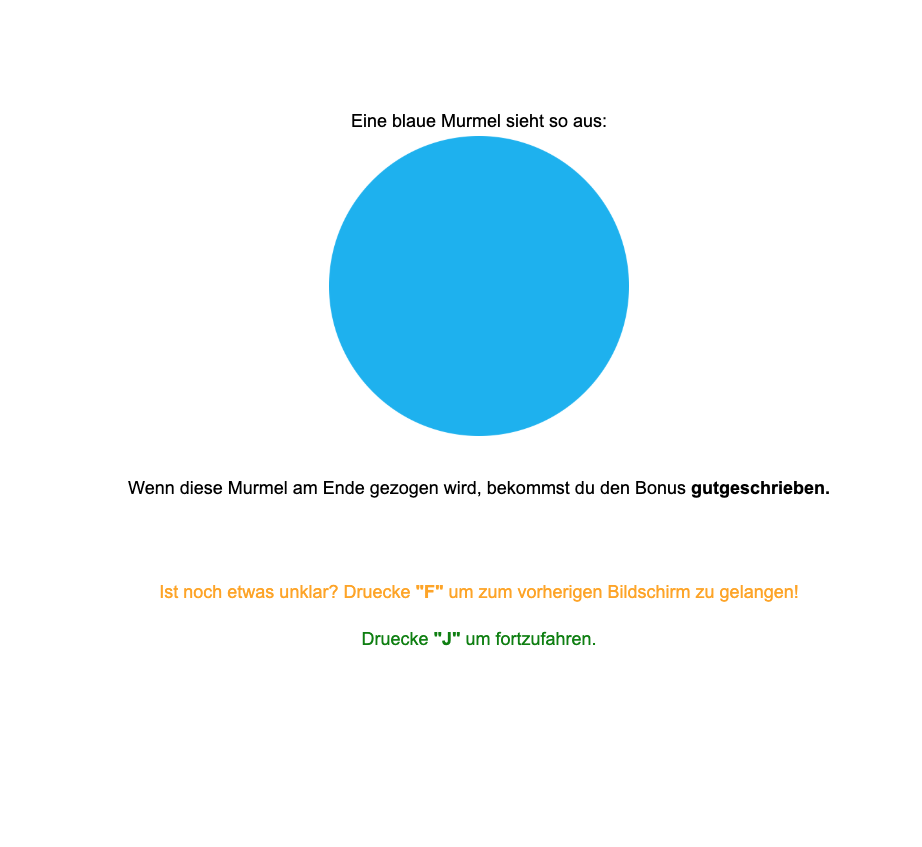


16.


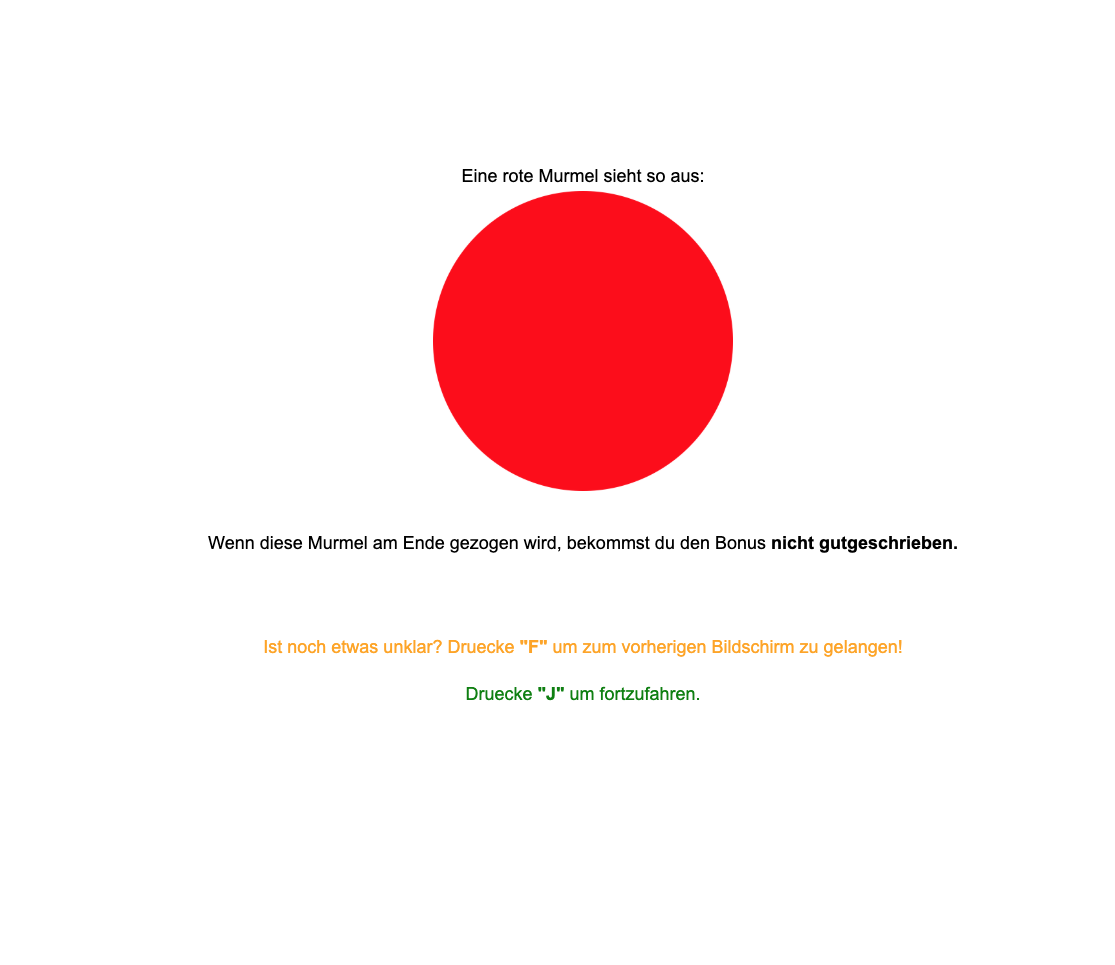


17.


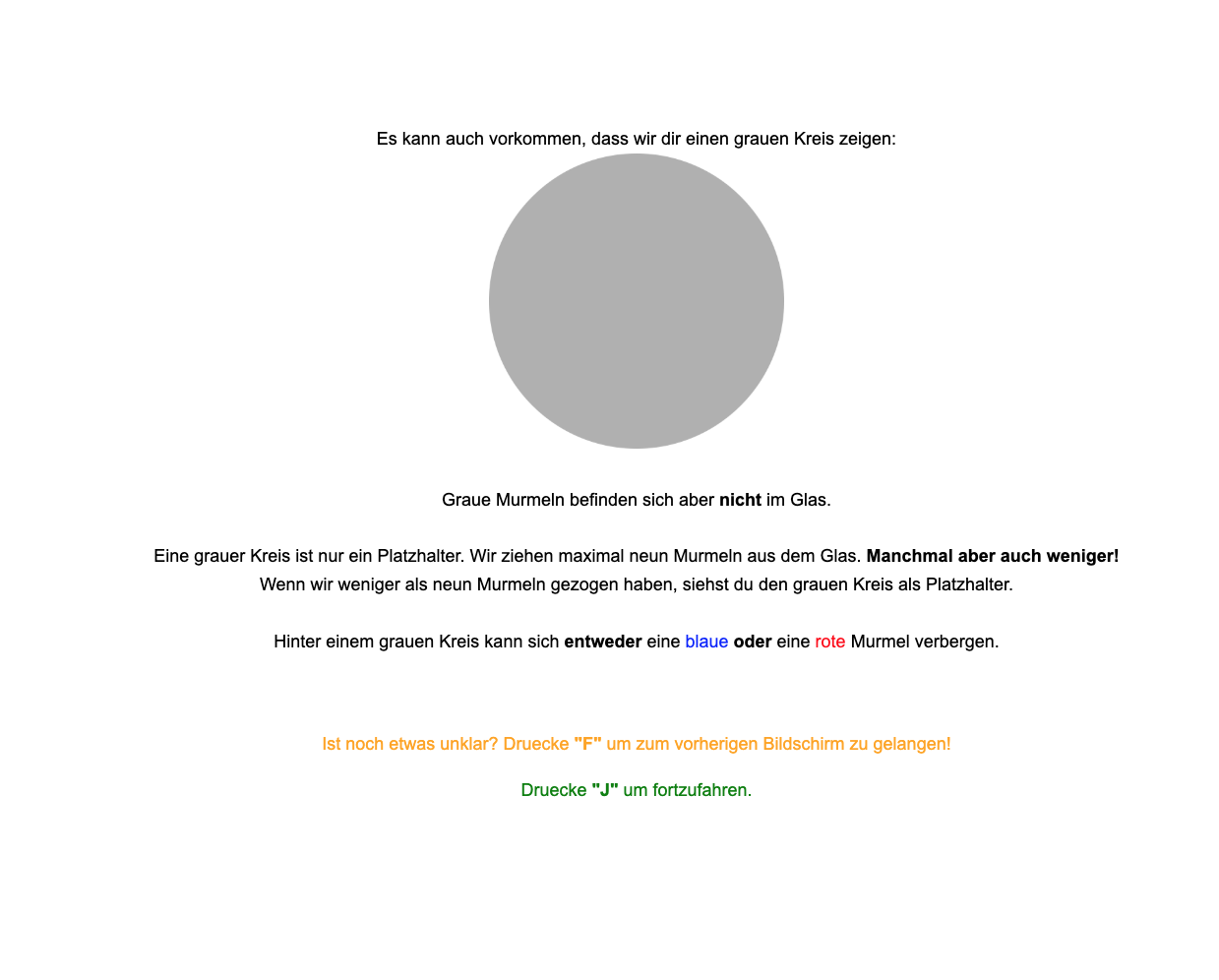


18.


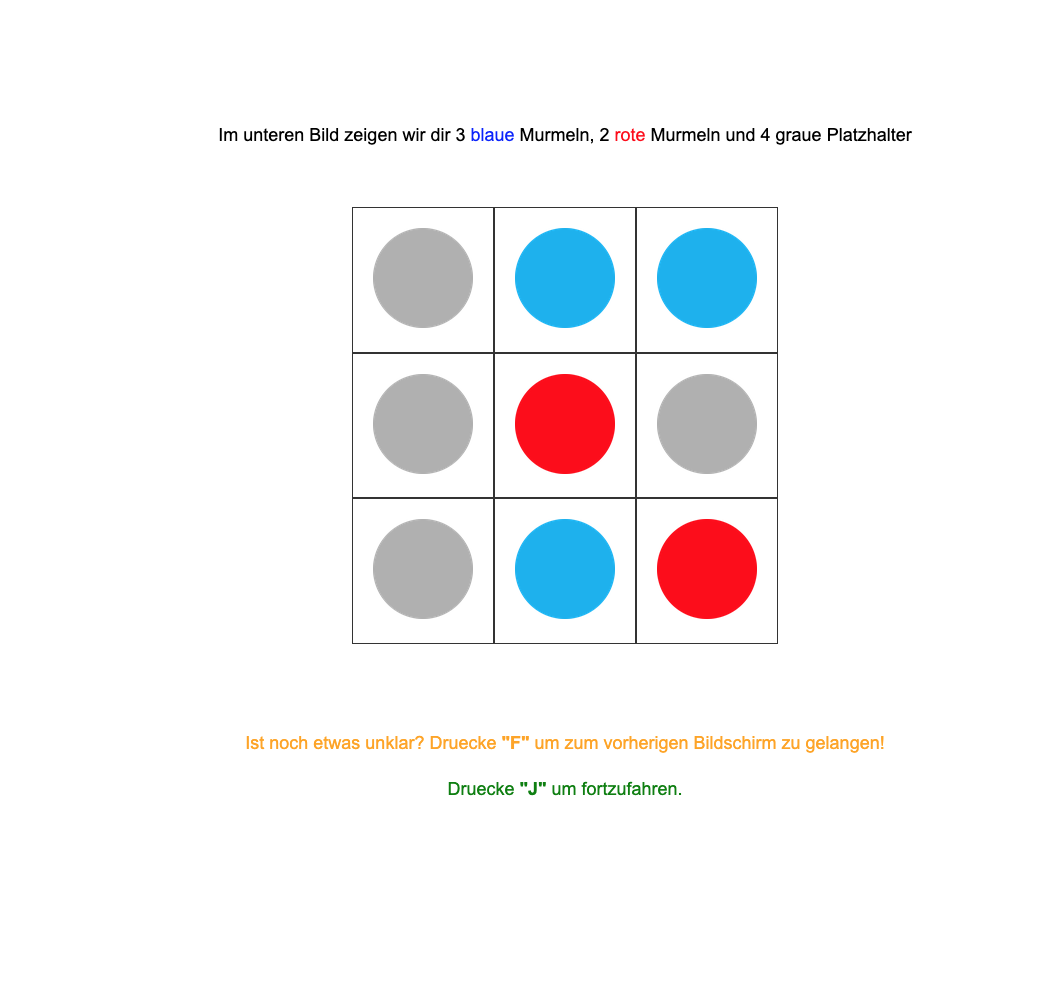


19.


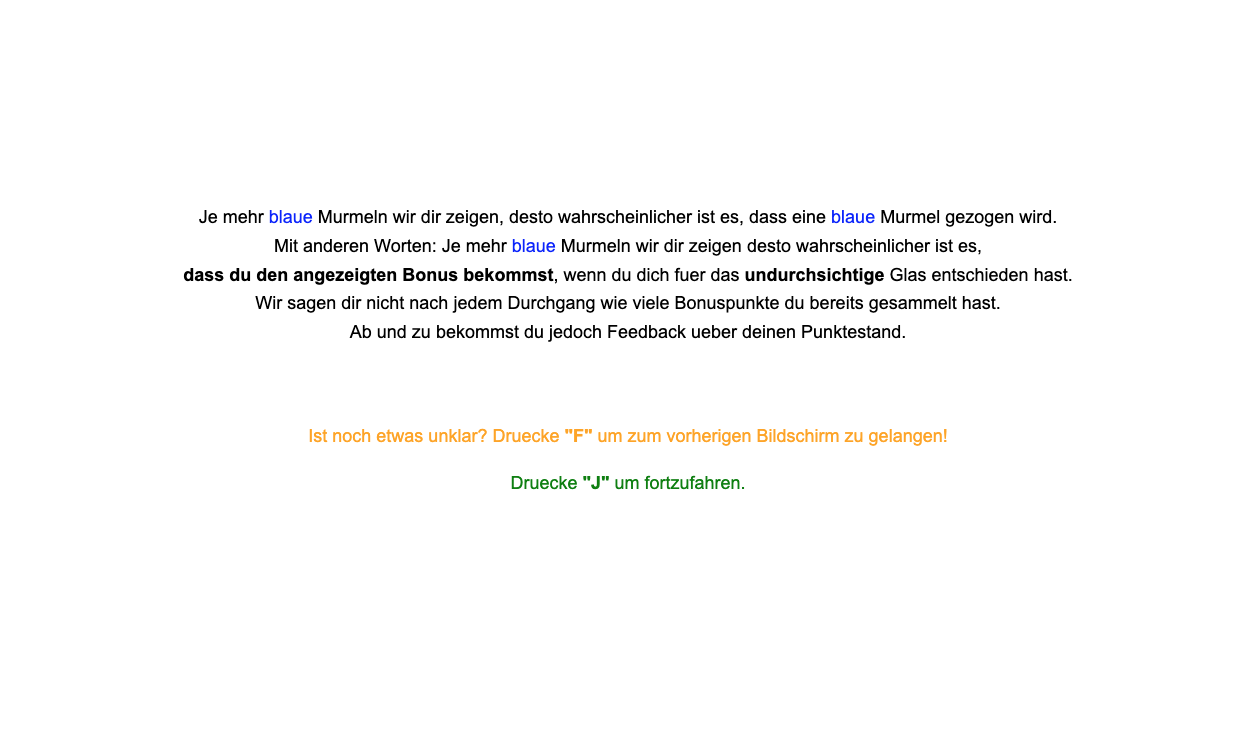


Comprehension Check 2

20.


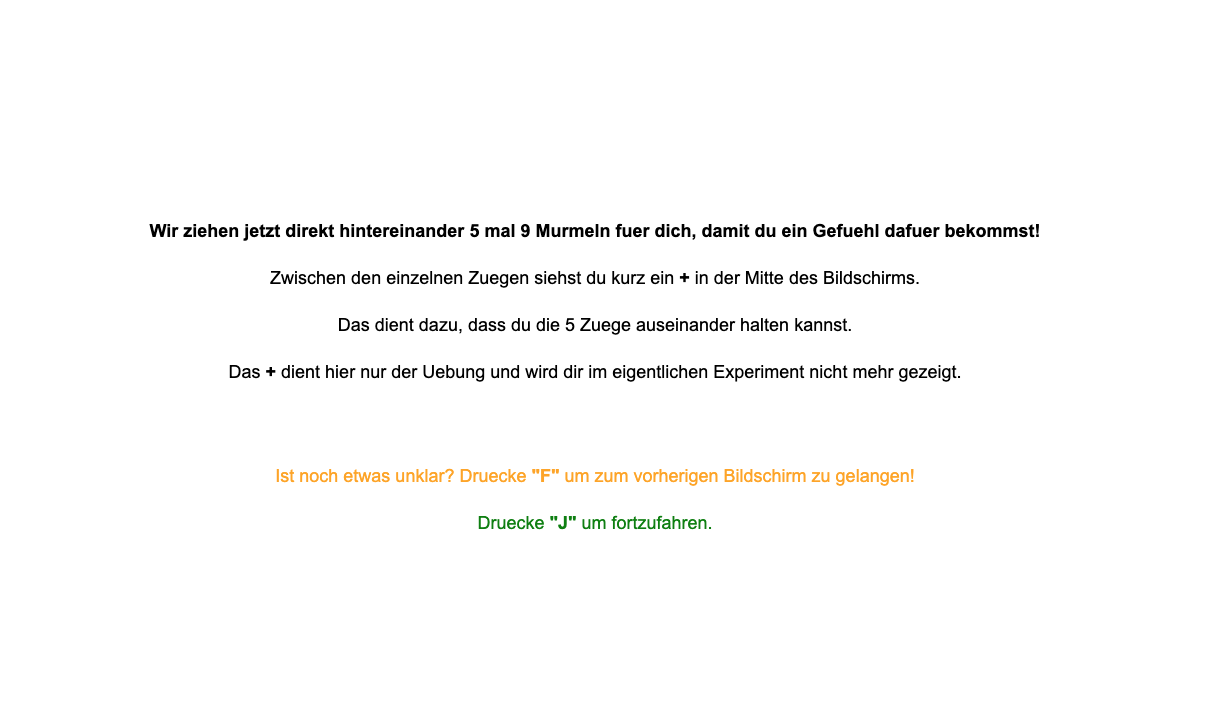


21.


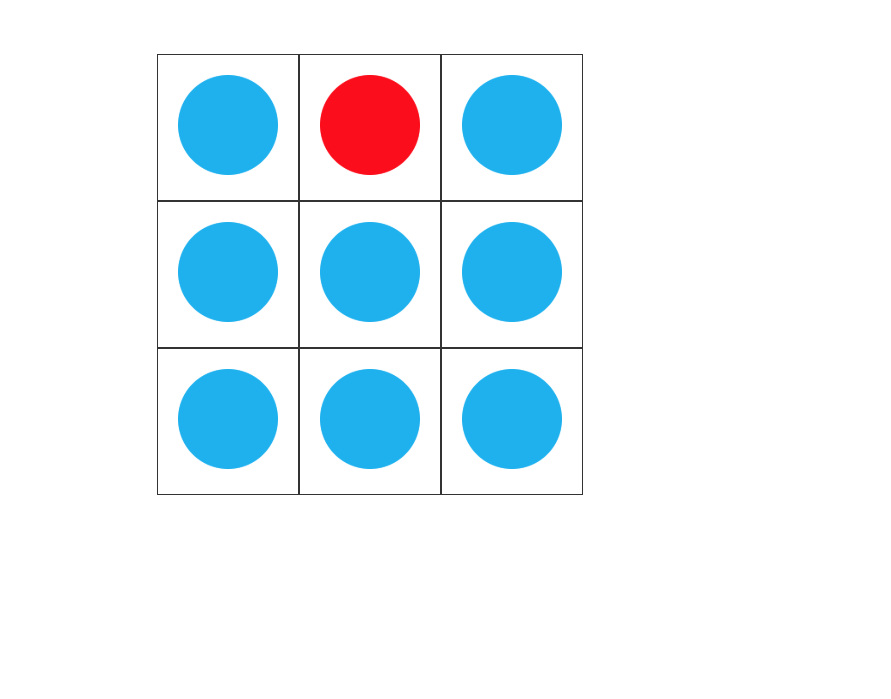
(5x)

21 a (wrong)


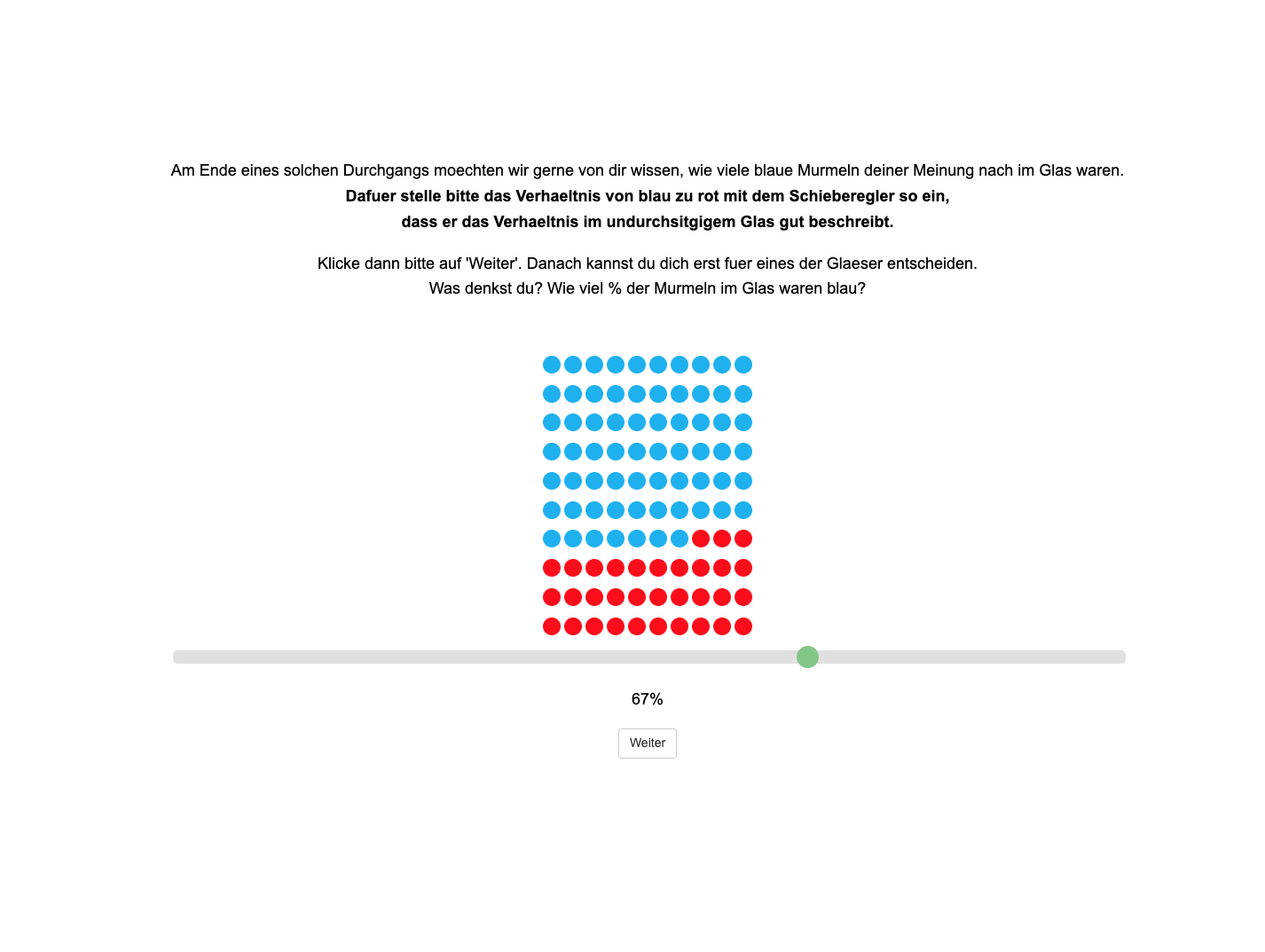


If wrong (response <80%, then back to 19)

22.


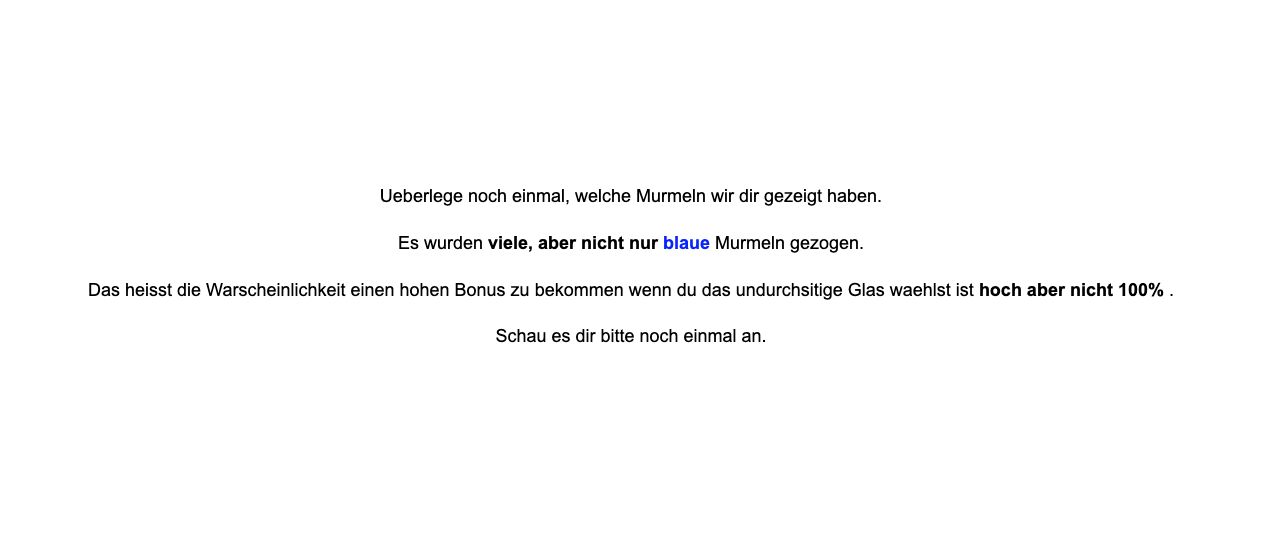


22 (right)


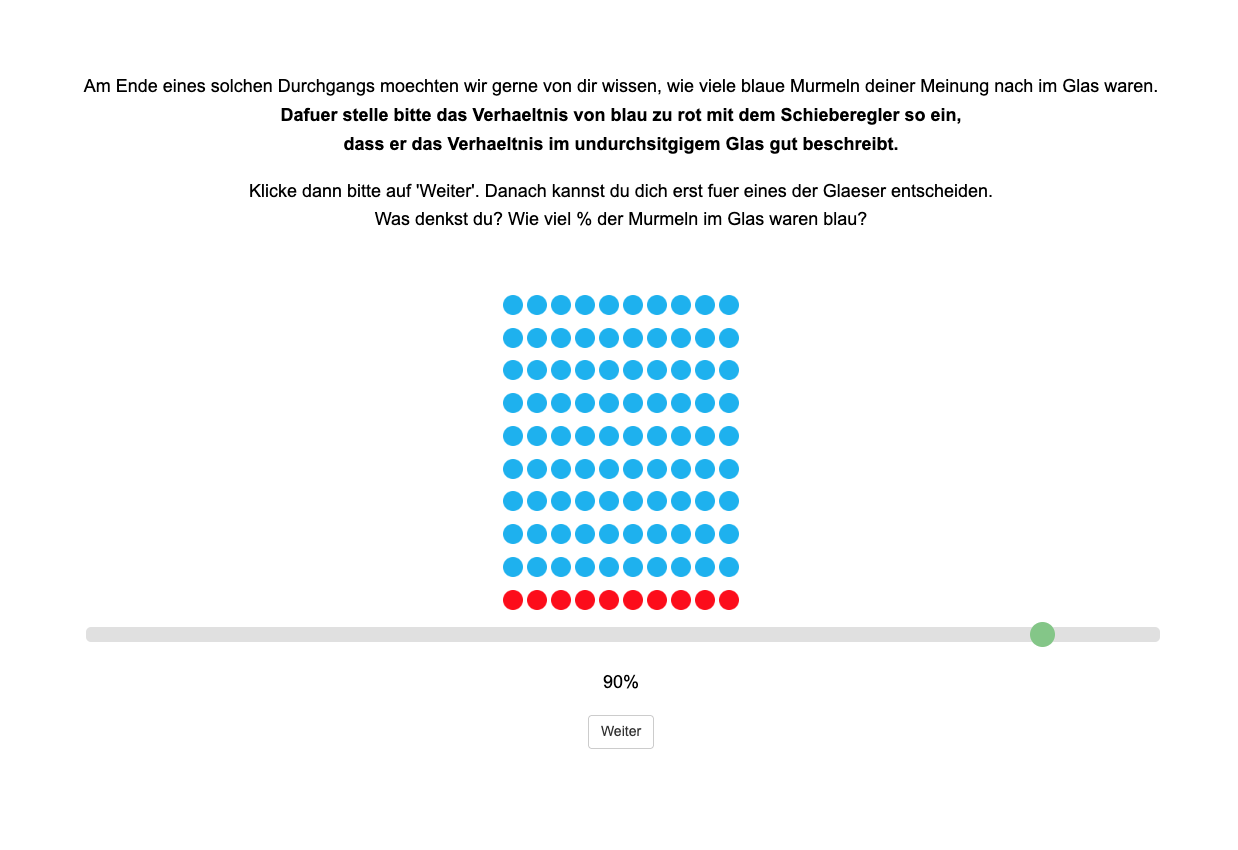


Comprehension Check 3

23


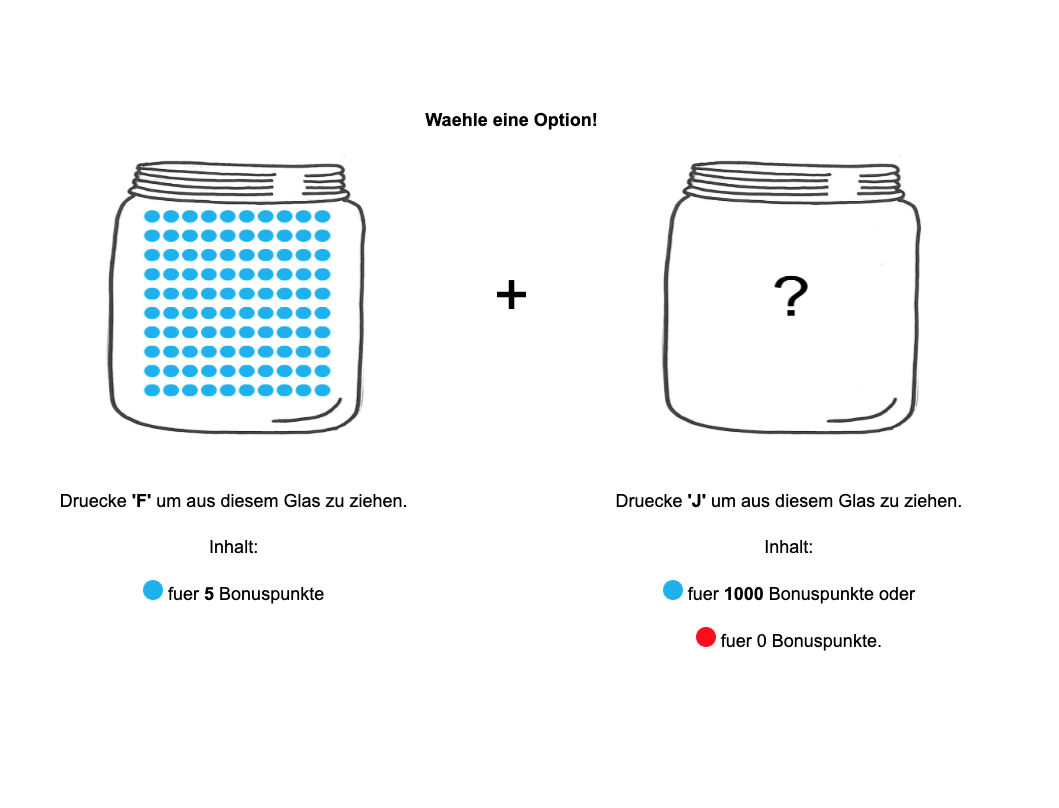


24 a If wrong (safe choice, then back to 23)
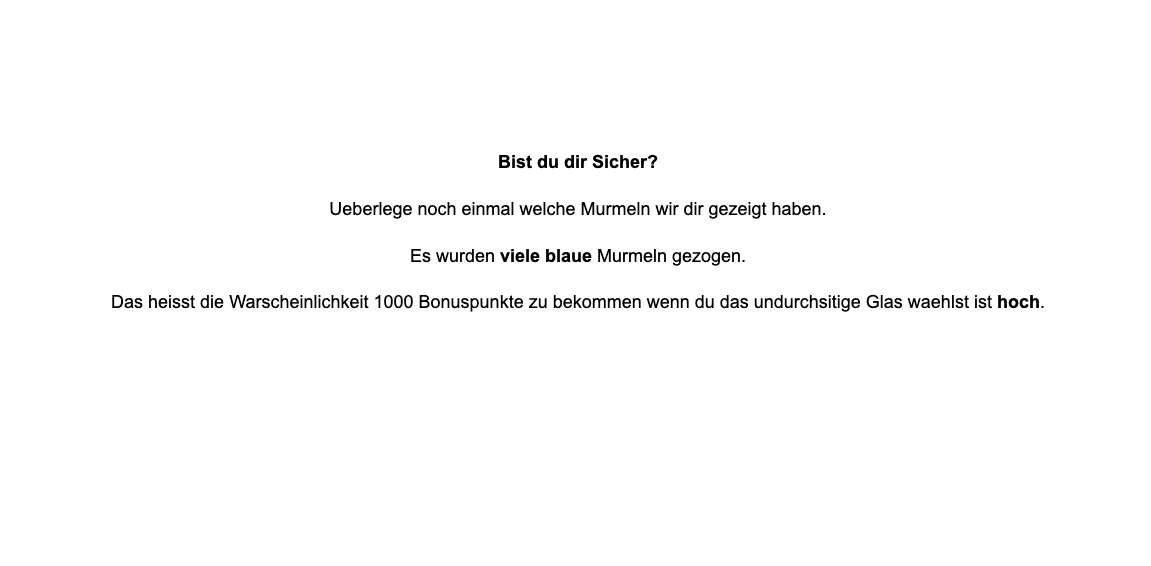


24 b If correct (risky choice)
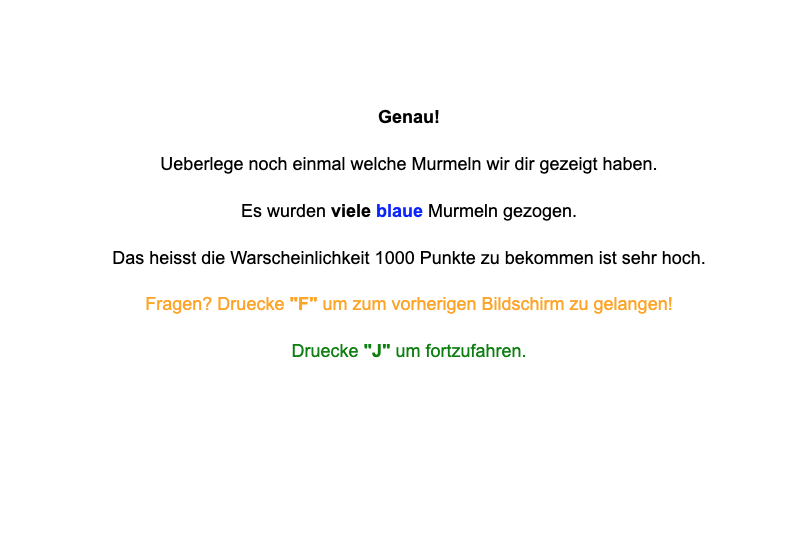


25


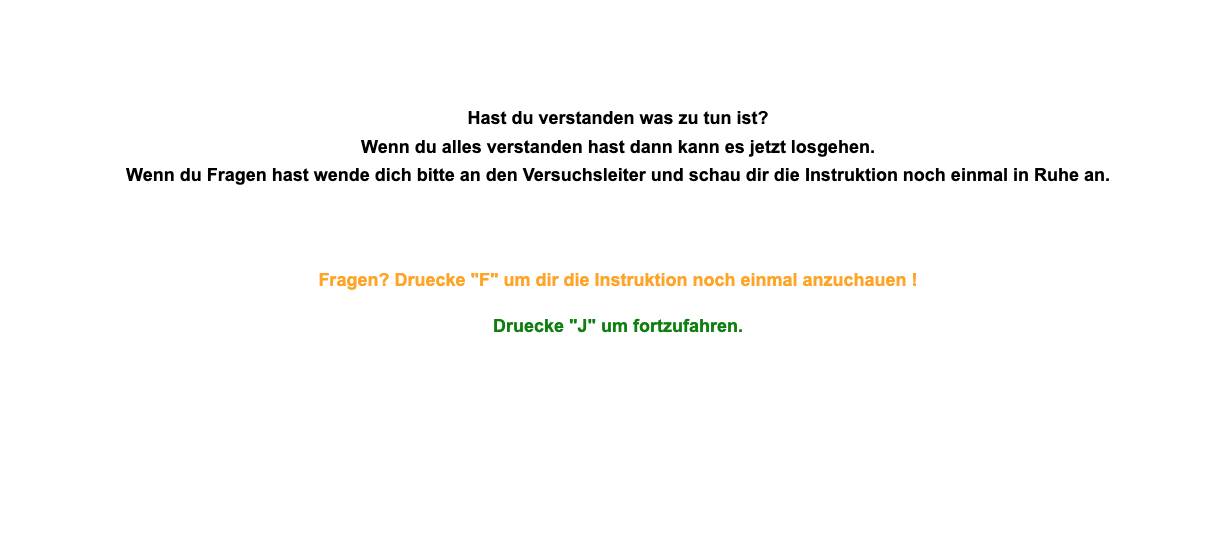


26


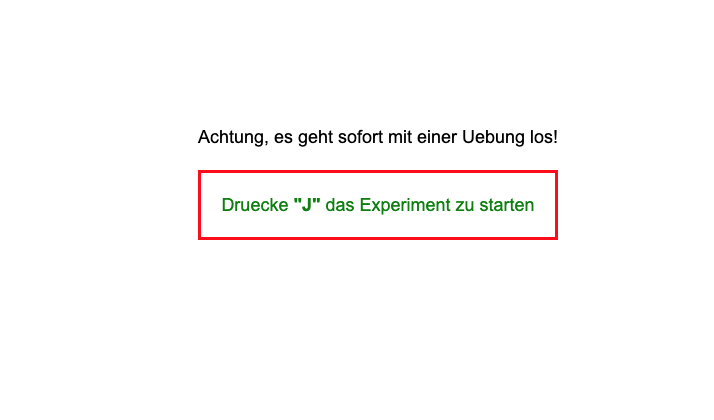

Supplement: Supplementary file 2 — Internal uncertainty impacts social information use in risky choice across adolescence. Supporting Information [file 44271_2025_314_MOESM2_ESM.docx]
